# Variations in near-infrared spectroscopy-derived oxygen downslope during a vascular occlusion test in critically ill patients: relationship with outcome

**DOI:** 10.1186/cc14221

**Published:** 2015-03-16

**Authors:** A Donati, E Damiani, A Carsetti, V Monaldi, E Montesi, P Pelaia

**Affiliations:** 1 Università Politecnica delle Marche, Ancona, Italy

## Introduction

Near-infrared spectroscopy (NIRS) with a vascular occlusion test (VOT) can be used to extrapolate information regarding the tissue oxygen extraction rate. We explored the meaning of variations in tissue oxygen saturation downslope (StO_2_down) during a VOT in critically ill patients.

## Methods

In this prospective observational study, NIRS (thenar eminence) was applied every day in 93 patients admitted to the ICU. A VOT was performed using a 40% StO_2_ target. The slope of the desaturation curve was assessed separately for the first part (StO_2 _down1) and the last part (StO_2_ down2) of the curve and the difference between Down2 - Down1 was calculated.

## Results

**Figure 1:**
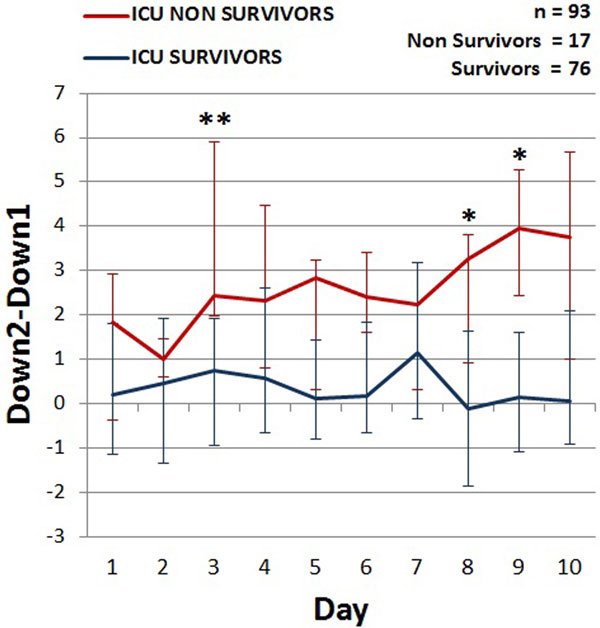


No significant differences were seen in StO_2_ Down1 or Down2 between ICU survivors (*n *= 76) and ICU nonsurvivors (*n *= 17) over the first 10 days in the ICU, while Down2 - Down1 was higher in ICU nonsurvivors (Figure 1). Patients in the upper quartile of mean Down2 - Down1 showed the highest 90-day mortality (*P *= 0.014).

## Conclusion

ICU nonsurvivors tended to show a flattening in the last part of the desaturation curve during a VOT, suggesting a reduced tissue oxygen extraction. This may depend on microvascular dysfunction and/or cellular hypometabolic status.

